# Monitoring Antioxidant Consumption and Build-Up in Polypropylene During Open-Loop and Closed-Loop Mechanical Recycling

**DOI:** 10.3390/ma18071640

**Published:** 2025-04-03

**Authors:** Niek Knoben, Max Vanhouttem, Aike Wypkema, Nithya Subramanian

**Affiliations:** Brightlands Materials Center, Urmonderbaan 22, 6167 RD Geleen, The Netherlands; niek.knoben@tno.nl (N.K.); max.vanhouttem@tno.nl (M.V.); aike.wypkema@tno.nl (A.W.)

**Keywords:** antioxidants, mechanical recycling, oxidative degradation, closed-loop recycling, open-loop recycling, polypropylene

## Abstract

Polypropylene (PP), a widely used recyclable plastic in packaging and engineering applications, is prone to thermo-oxidative degradation during reprocessing and molding at high temperatures. Antioxidants (AOs) are essential for stabilizing PP in both its virgin and recycled states. The quantity of AO added is critical: insufficient amounts can lead to poor stabilization, while excessive amounts can cause safety concerns due to build-up. This study presents a modified approach to measure the Oxidation Induction Temperature (OIT) using Differential Scanning Calorimetry (DSC), particularly for recycled PP from waste that contains unpredictable contaminations. This modified approach ensures the safety of the calorimetry cell by limiting the oxidation reaction and preventing the release of volatile compounds during measurements. By performing DSC measurements in inert environments, we obtain the OIT, which can be correlated to residual intact AO levels. This approach to monitoring AO levels is demonstrated in both open- and closed-loop recycling of rigid PP. Although the presence of contamination is known to catalyze thermo-oxidative degradation in PP, our results indicate that recycled PP from open-loop collection still contains sufficient residual AO that allows it to withstand limited thermal reprocessing. However, this tendency of AO retention leads to significant build-up during closed-loop recycling when AOs are added to each cycle, where the PP grade remains fairly homogeneous and the dispersity (Đ) does not significantly increase over multiple recycling loops.

## 1. Introduction

The increase in demand for plastics has led to an exponential increase in production, which contributes to an accumulation of waste generated annually, as well as the over-usage of associated resources [1,2,3,4]. Both the waste generated and the over-usage of resources pose significant problems to society, for instance, by contributing to high CO_2_ emissions and climate change, as well as biodiversity loss from practices such as waste landfilling. Urgent solutions are necessary to tackle these problems [5,6]. Mechanical recycling is a widely used method to reduce the reliance on fossil-based virgin plastics and recover materials from plastic waste. However, the suitability of recycled plastics from mechanical recycling for sensitive applications (e.g., food or cosmetic packaging) is an area where technological innovation in decontamination needs to be advanced to ensure regulatory compliance [7]. It is necessary to investigate these problems arising from the quality and performance degradation of polymers to make mechanical recycling a more feasible option for a wider range of products and packaging applications.

The packaging industry represents one of the biggest sectors of plastic usage and generates almost half of the annually produced plastic waste worldwide [8]. As of 2021, only 41% of plastic packaging waste enters recycling in Europe, from which significant material is lost during the recycling process [7,9,10,11]. Polyethylene (PE) and polypropylene (PP), often referred to as polyolefins, account for almost 70% of the plastic packaging market in Europe [12]. These polyolefins are used for numerous different packaging applications, ranging from food and beverages to personal care products [13]. Upon mechanical recycling, the quality of these polyolefins drops due to the degradation of the polymer.

**Figure 1:**
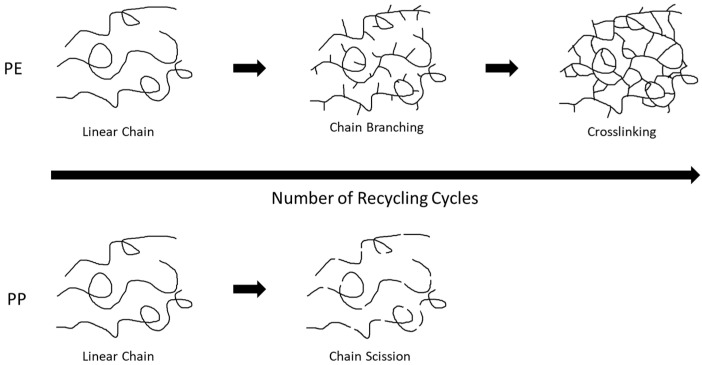
Degradation mechanisms during recycling in polyolefins. PE chains undergo branching and crosslinking among each other, whereas, in PP, the cutting down of chains in length more commonly occurs.

Depending on the type of polyolefin, different degradation behaviors can occur, as shown in Figure 1 [14]. The different types of degradation—chain-branching, cross-linking, and chain-scission—influence the properties of the polymers, such as viscosity and tensile strength, which is most often undesirable from processability and functionality perspectives. [14]. Polymers can be protected from different external factors that contribute to degradation, such as ultraviolet (UV)-radiation and heat, by adding additives to the polymerization or (re-)compounding step [15]. To prevent chain-scission in PP specifically, antioxidants (AOs) can be used, which are added in the compounding step of the majority of commercially available PP grades and often in the re-compounding step upon recycling [7,16].

**Figure 2:**
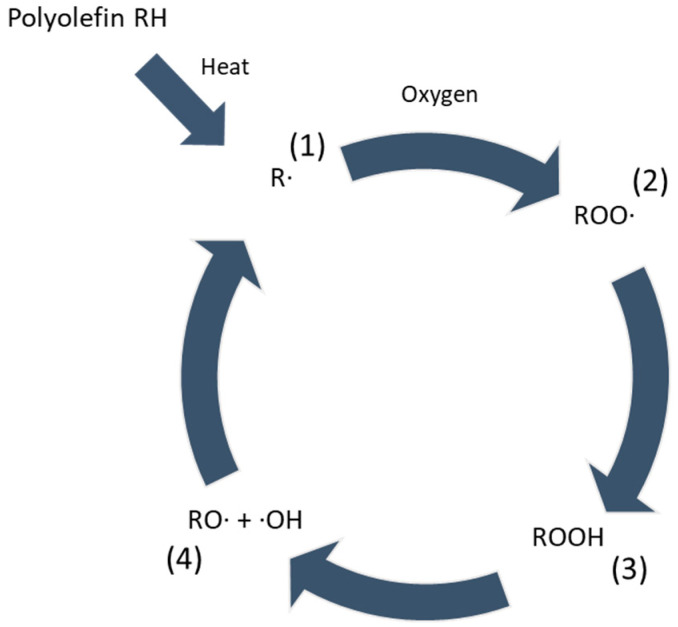
Thermo-oxidative degradation steps in PP resulting in chain scission and overall shorter chain lengths.

Degradation in PP often occurs due to the polymer being exposed to high shear forces and high temperatures in the presence of oxygen during extrusion processes, often referred to as thermo-oxidative degradation [17,18]. Within this common degradation scheme, radicals are formed in multiple steps due to the presence of heat and oxygen, as illustrated in Figure 2, where R depicts the backbone of the polyolefin [18]. The presence of radicals leads to the formation of end groups, and thus the end of a polymer chain, resulting in shorter chains, known as chain scission, as depicted in Figure 1 [14,18]. Molecular Weight Distribution (MWD) of the PP also influences the degradation process as higher-molecular-weight chains can typically resist the onset of degradation due to more entanglements, thereby slowing down the diffusion of oxygen and mobility of free radicals [19]. However, once the degradation starts, higher-molecular-weight chains undergo chain scission more readily. Thus, a broad MWD with the presence of both low- and high-molecular-weight chains can cause the short chains to degrade faster and the long chains to resist initial degradation, creating an interplay effect. The presence of short-chain branching also plays a role as these branches create more routes for exposure to oxygen and sites for the initiation of degradation.

To prevent the formation of radicals in the backbone of the polymer chain, primary antioxidants, which are often sterically hindered phenolic chemicals, are added. These phenolic antioxidants inhibit radicals by acting as radical scavengers and neutralizing them before they can initiate the degradation process, thus decelerating chain-scission [18]. Besides the thermo-oxidative degradation occurring during processing, the PP backbone can also be degraded during the use phase, resulting in a reduction in important mechanical properties [17,18,20,21]. Primary AOs also suppress oxidative degradation during the use phase, maintain the polymer’s integrity, and extend its service life. Equations (1) and (2), which correspond to (1) and (2) in Figure 2, depict how the radical scavengers act to protect the polymer, where *A_RS_* represents the primary AO.

(1) $$R\cdot +A_{RS}\;H\to RH+A_{RS}\cdot$$

(2) $$ROO\cdot +A_{RS}\;H\to ROOH+A_{RS}\cdot$$

Synergistically with the primary AO, secondary AOs, which are often phosphites, are also added during processing [18]. Secondary AOs decompose hydroperoxides into stable products and are therefore also referred to as peroxide decomposers. Hydroperoxides are formed during the oxidation process and can further decompose into free radicals if not neutralized. Equation (3), which corresponds to (3) in Figure 2, shows how the peroxide decomposers function, where *A_PD_* represents the secondary AO.

(3) $$ROOH+A_{PD}\to ROH+A_{PD}O$$

By breaking down hydroperoxides, secondary antioxidants prevent the formation of new free radicals, thus supporting the primary antioxidants [22]. Secondary AOs play a critical role in the processing phase, whereas primary AOs offer protection both during the processing and use of the polymer. Due to this synergistic cooperation, the possibility of the product formed in (4) in Figure 2 is lower. Consequently, the likelihood of propagation of radicals is mitigated, leading to a deceleration of chain-scission in the PP backbone.

Recyclers often add primary and secondary AOs to achieve synergistic protection against thermal degradation, in addition to processing aids, during the recompounding of used polyolefins. If an excess of AO is added in each recycle processing step, a build-up of AOs (and their breakdown products) might occur, which can have implications on the safety of using recyclates in packaging applications, especially in food packaging due to risks of migration [23,24]. Determining the amount of active and degraded AO in the polymer matrix is necessary to determine whether new AOs need to be added during the next recycle processing loop. However, determining these amounts is normally accomplished with expensive and time-consuming analytics such as High-Performance Liquid Chromatography (HPLC), Reverse-Phase Liquid Chromatography (RPLC), and Gas Chromatography (GC). Recyclers often forgo this analysis owing to the prohibitive nature of sample preparation required such as extraction of the antioxidants subsequent to dissolving the polymer and the cost of the analytical processes [25,26,27]. One way to determine the presence of AOs in a qualitative but useful way is by calculating the Oxidation Induction Temperature (OIT) and Oxidation Induction time (OIt) with Differential Scanning Calorimetry (DSC)—a thermal characterization that is inexpensive and easy to perform without intensive sample preparation steps [28,29]. An oxidation-protected polymer oxidates at a higher temperature and a later time when determining OIT and OIt, respectively, compared to the same polymer in an unprotected or less protected state [29]. When a build-up of AO occurs due to the addition of AOs in each compounding step, the OIT and OIt would further increase.

The open-loop mechanical recycling process reflects current practices where plastic waste, collected through both mixed and separate collection systems across various municipalities, is sent to a recycling facility without distinguishing the source or intended application [30]. However, the European Food Safety Authority (EFSA) approval for recycled materials in food-contact applications requires a shift toward closed-loop recycling [31]. In this study, we define open-loop recycling as the treatment and processing of waste from mixed and separate collections that are sorted into an assigned stream. DKR is a German abbreviation used to specify all waste streams and is used by recyclers in different European countries, such as The Netherlands, to specify waste streams sorted in their facilities. The composition and properties of these streams are often uncertain/inconsistent and may include contaminants and minor compositions of non-target polymers. The MWD of the target polymer found in these waste streams is generally broad as it contains a variety of polymer grades used in a wide range of processing applications, such as injection molding, thermoforming, blow molding, fiber extrusion, etc.

In contrast, closed-loop recycling involves collecting waste, such as PP, from a single application with clearly identified grades and reprocessing it through multiple cycles to return it to the same application [32,33]. Within this paper, our goal is to demonstrate the use of DSC to determine the AO content in a qualitative way with the OIT in post-consumer open-loop recycling and post-consumer multiloop closed-loop recycling. With this technique of determining AO content within post-consumer recyclate batches with DSC, a constructive decision can be made on the need to add AOs within a follow-up processing step. Furthermore, an indication of the amount of AO present in recyclates can offer insights into preventing the build-up of AOs and ensuring better safety of recycled materials.

## 2. Experimental

### 2.1. Materials

For the open-loop part of this study, DKR-324 was selected as the waste stream and was obtained from a Dutch recycling company. DKR-324 is a post-consumer rigid PP waste stream that consists of at least 94% PP, with the remaining 6% being other waste material [34]. In general, it contains few flexibles and has a Melt Flow Rate (MFR) of ~21 g/10 min (230 °C, 2.16 kg). Four different DKR-324 batches were identified for this research and are named in accordance with their sorting specifications: Mixed, White, Transparent, and Color. Sorting was performed on the Mixed fraction based on the color of the articles and, subsequently, the color of the flakes to obtain White, Transparent, and Color. The post-sorted fraction named Color consists of mass-colored flakes in the waste stream. The fraction titled Mixed is the original DKR-324 as is, without undergoing any post-sorting for color. All DKR-324 batches were obtained in quantities of 10 kg, subsequent to near-infrared sorting, shredding, and alkaline hot-washing of flakes.

For the closed-loop recycling scheme, an injection molding PP grade with an MFR of 44 g/10 min was selected. This PP grade was obtained from injection-molded buckets used in restaurant kitchens for condiment packaging. For this study, new buckets (without any food contamination) were considered. Two AOs were selected for this study, namely primary AO Irganox 1010 (pentaerythritol tetrakis [3-[3,5-di-tert-butyl-4-hydroxyphenyl]propionate, CAS number: 6683-19-8) and secondary AO Irgafos 168 (tris (2,4-di-tert.-butylphenyl) phosphite, CAS number: 31570-04-4), procured from Sigma-Aldrich (Buchs, Switzerland), and manufactured by BASF (Moerdijk, The Netherlands).

The virgin PP used for validation was PP 310MK40 from SABIC Petrochemicals (Geleen, The Netherlands), an impact copolymer grade used in injection molding applications.

### 2.2. Sample Preparation

In the open-loop recycling scheme, each post-sorted DKR-324 fraction was divided into two batches; one was compounded without adding any AO while the other batch was dry-blended in powder form with 500 ppm primary AO and 1000 ppm secondary AO and subsequently compounded. Both batches were injection molded to tensile bars according to ISO-527-1A [35]. It must be noted that the initial amount of AOs present in the original DKR-324 fractions may have differed but were not investigated in this study.

**Figure 3:**
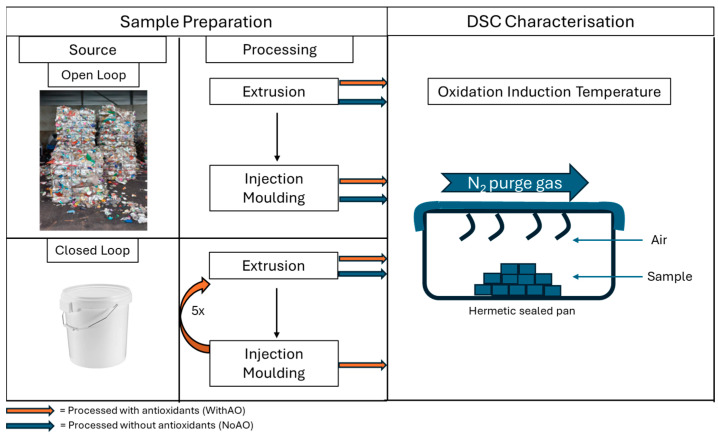
Schematic of the source materials, processing, sample preparation, and characterization performed in the study.

For the closed-loop recycling study, two separate batches were generated. In the first batch, the PP grade was compounded with AOs, subsequent to dry blending, and then injection molded to tensile bars according to ISO-527-1A. Untested tensile bars were shredded and prepared for the next extrusion cycle. This entire process was repeated five times to simulate a multiloop recycling scheme, as illustrated in Figure 3. As per the compounding step, 500 ppm of the primary AO was dry-blended, while 1000 ppm of the secondary AO was dry-blended. In the second batch, the PP grade was compounded without AO five times, skipping the injection-molding step in between. This is mainly because the processability of PP without AOs became increasingly challenging with each reprocessing cycle. Adding an extra thermal step via injection molding in each cycle would have made it impossible to complete the study through all five loops.

Compounding was performed on an APV Baker Industrial MP19PC (Baker Perkins Ltd., Peterborough, UK), (25:1 L/D ratio) twin-screw extruder with co-rotating screws at a screw speed of 150 rpm and a degassing vent along the barrel. The conformation of the screws exists in three kneading zones to increase mixing. The temperature profile was 190 °C (hopper) to 230 °C (die), with a residence time of 20 s. The closed-loop batch without AOs was compounded on an Xplore MC 15 HT micro-compounder (Xplore Instruments BV, Sittard, The Netherlands), with a 15 mL capacity due to the limited material available. Micro-compounding was performed with a reflux channel, with the temperature set to 230 °C and a total residence time of 20 s. Injection molding was carried out on Engel Victory 50 with a temperature profile of 190 °C to 230 °C. Residence time in the barrel was 25 s, with a subsequent injection time of 1 s, and finally, a cooling cycle at a temperature of 60 °C that lasted 25 s.

### 2.3. Characterisation

#### 2.3.1. Thermal Analysis

This study considers two main thermal characteristics to determine the consumption of AOs during processing, namely the Oxidation Induction Temperature (OIT) and the Oxidation Induction time (OIt), both determined with DSC on TA Instruments Discovery DSC 2500 (TA Instruments, Hüllhorst, Germany). Conventionally, the OIT is determined by exposing the sample to airflow while being heated in a DSC. This often yields a large and sometimes noisy oxidation peak, implying an oxidative reaction in the PP backbone. In our study, the OIT is calculated by performing a heating cycle (temperature sweep at 10 °C/min) with a hermetic pan with nitrogen as flow gas, as illustrated in Figure 3. The amount of oxygen trapped in the hermetically closed pan is the limiting factor for oxidation, which will therefore give a smooth peak. Furthermore, our approach ensures that the volatiles formed during the degradation process, especially from DKR-324 (due to the unpredictable contaminants in waste flakes), are contained within the hermetically sealed pan. This containment protects the DSC cells from the risk of corrosion. Additionally, the reaction is stoichiometrically limited by the oxygen already trapped in the sealed pan, and this oxidation peak will not appear in the second heating cycle. Three replicates were measured per batch to measure the OIT.

The OIt is calculated using a traditional approach by heating the sample, which is placed in a pan with a normal lid, at 50 °C per minute, with nitrogen used as flow gas to create an inert atmosphere. After reaching the required temperature, the gas is switched to air and the temperature is kept isothermal for a fixed amount of time. Since this approach poses a risk of corrosion to the cell, only one measurement per sample is carried out to measure the OIt, yielding no standard deviations for this measurement. Besides DSC, the MFR is measured to obtain an indication of the effect of the AOs on the degradation of the PP. MFR is obtained according to ISO 1133, with a temperature of 230 °C and a weight of 2.16 kg, measured on granulates with ZwickRoell Mflow (ZwickRoell, Venlo, The Netherlands) [36].

#### 2.3.2. Chemical Analysis

Size Exclusion Chromatography (SEC) is performed on samples using a universal calibration method on Polymer Laboratories PL-GPC220 (Agilent Technologies Netherlands B.V., Middelburg, The Netherlands), using a refractive index detector to determine the MWD in order to obtain more information about the degradation of the backbone of the PP. The solvent, 1,2,4-trichlorobenzene, is used to dissolve PP at 160 °C. The column set used consists of three Polymer Laboratories 13 µm PLgel Olexis, (Agilent Technologies Netherlands B.V., Middelburg, The Netherlands), 300 × 7.5 mm. PE molar mass calibration was performed with linear PE standards. PP molar mass calibration was obtained after conversion from PE to PP using the Mark–Houwink constants of PE and PP. HPLC with diode-array detection (DAD) was performed to quantify the amount of active and degraded AOs to back up the DSC results. The column used for HPLC was Agilent Poroshell 120 C18 (Agilent Technologies Netherlands B.V., Middelburg, The Netherlands), 50 mm × 4.6 mm × 2.7 µm, and the mobile phase was water/acetonitrile in a 70/30 ratio with a runtime of 30 min. A retention time of 21.3 min was used to identify intact Irgafos 168 and 16.2 for Irgafos 168 that had been consumed. Similarly, a retention time of 13.9 min was used to track active Irganox 1010.

## 3. Results

### 3.1. Open-Loop Recycling

The results in this section primarily identify the composition of PP obtained from DKR-324, its post-sorted fractions (Mixed, White, Transparent, and Color), and their tendency to undergo oxidative degradation.

#### 3.1.1. Composition

**Figure 4:**
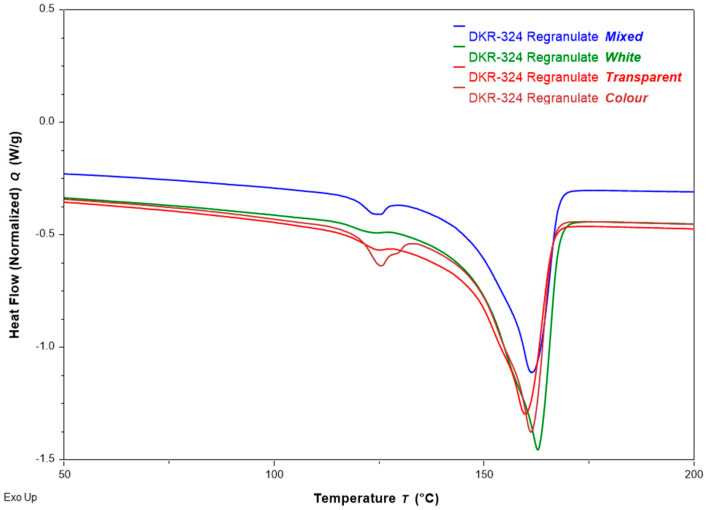
DSC heating curves for post-sorted DKR-324 fractions showing PE and PP compositions. The mass-colored fraction appears to contain the highest PE fraction, whereas the white flake-sorted fraction has the lowest PE fraction. The cross-contamination from PE could arise from rigid PP packaging articles containing elements that are made up of HDPE (e.g., PP bottle with HDPE cap) or from flexible PE labels/lidding films.

It has been known for a long time that DSC measurements can shed light on the composition of blends and also help estimate/quantify the make-up of a material blend. Recently, a machine-learning approach was implemented on the DSC curves of blends to estimate the composition more accurately [37]. Figure 4 overlays the heating curves of all regranulates from four DKR-324 fractions and illustrates the presence of two distinct peaks corresponding to PE and PP in the mixture. This finding is not surprising given that the specifications for this sorted stream indicate a purity level of approximately 94% PP in the Netherlands and around 96% PP in Belgium [38]. The primary polymer present in the remaining fraction is polyethylene (PE), which includes rigid high-density polyethylene (HDPE), flexible linear low-density polyethylene (LLDPE), and low-density polyethylene (LDPE). The rigid HDPE likely originates from mixed-material packaging, such as PP bottles with HDPE caps, while the LLDPE and LDPE most likely originate from the labels and sleeves on packaging. Additionally, ethylene may be present because some PP grades are copolymerized with ethylene to enhance impact resistance. As a result, many packaging materials requiring improved impact strength contain a certain amount of this ethylene in the copolymer phase. However, this is too small to be detected via our DSC methodology.

From the DSC heating curves, we estimate the following proportions of PE in the four chosen DKR-324 fractions: 1.8% in Mixed, 0.3% in White, 1.6% in Transparent, and 5.9% in Color. The measured compositions are logical as they align with current packaging trends in the market. White fractions, typically used in dairy applications with in-mold labels and take-out containers, are made from injection molding-grade PP. White PP packaging also comes with very few PE labels/films. Transparent PP, commonly used in salad and vegetable packaging, could have some LDPE lidding films attached to it. The Color fraction generally originates from PP bottles and jugs, which often have HDPE lids or caps. Since sorting is performed at the article level first, there is a higher chance of these caps and lids ending up in the DKR-324 stream, thus yielding a relatively higher PE content in the Color fraction.

The MWD of the different color-sorted fractions (regranulates) are presented in Table 1. The Mixed fraction exhibits the highest dispersity (Đ) as it contains a mixture of all rigid PP articles from various sources. The White fraction is made up of relatively lower molecular weight (Mw) PP as it arises from injection-molded PP grades and possesses the lowest Đ [39]. Transparent PP packaging is generally thermoformed and therefore contains more Mw.

#### 3.1.2. Oxidative Degradation

**Figure 5:**
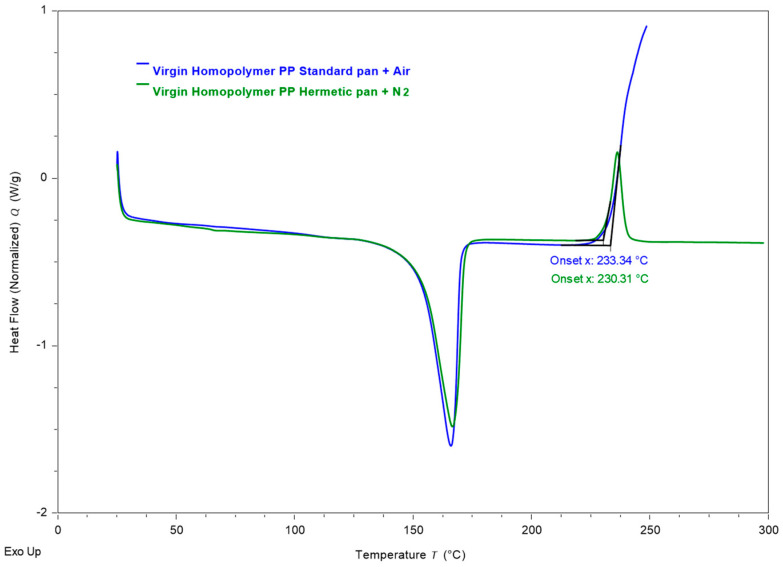
DSC heating curves showing the melting and oxidative degradation phenomena. In the standard pan, the oxidative degradation is initiated and progresses significantly due to the abundance of air. In the hermetic pan, the oxidative degradation is triggered by the air trapped in the sealed pan reacting with the PP backbone. The onset point of the oxidation peak is of interest to determine the initiation of the thermo-oxidative degradation process.

The heating cycles of virgin PP in the DSC measurements at a constant heating rate with a standard/open pan in the air and a hermetically sealed pan are illustrated in Figure 5. Beyond the typical key features pertaining to the melting of crystalline phases (around 160 °C for PP), we observe an additional exothermic peak as heating is extended to higher temperatures. This corresponds to the reaction of the air with the PP, leading to the oxidation of the polymer backbone. Typically, the characterization of this oxidation peak is conducted under an oxygen atmosphere (standard pan with air flow, shown in blue in Figure 5). In our study, the DSC measurements were performed in a hermetically sealed pan with nitrogen as the purging gas (shown in green in Figure 5). However, in the hermetic pan, a small amount of air is trapped during sample preparation in addition to some oxygen absorbed in the polymer itself. We demonstrate here that critical information about the initiation of oxidative degradation in the sample can be obtained from this limited-oxygen atmosphere. This method mitigates the generation of volatiles as byproducts of oxidative degradation and limits the exposure of the DSC cells to potential corrosive compounds of decomposition. Furthermore, the produced volatiles are contained within the hermetically sealed pan and the reaction ends when the trapped oxygen is depleted, yielding a complete peak. Therefore, we use this methodology and the information gathered to further analyze and compare the residual antioxidant levels in open-loop and closed-loop recycled PP samples.

The onset of the oxidation peak is identified and defined as the OIT [29,40,41]. OIT is a key metric used to evaluate the thermal stability and oxidation resistance of plastics. It represents the temperature at which a polymer, under a controlled environment, begins to rapidly oxidize when exposed to an oxygen-rich atmosphere. In our study, the atmosphere is not changed from inert to reactive. However, the PP becomes reactive to the small amount of oxygen/air trapped in the hermetic pan. The OIT depends on the concentration of antioxidants and their effectiveness. At temperatures beyond the peak maximum, the oxygen is completely depleted, rendering an end to the oxidative reaction in the pan.

**Figure 6:**
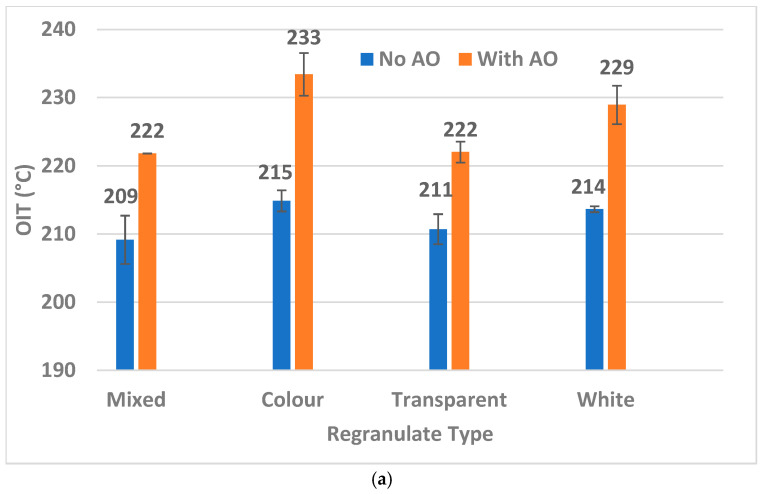

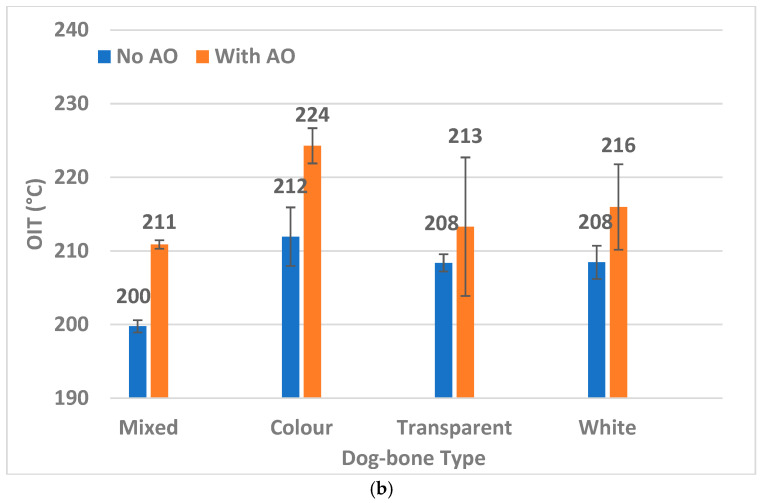
Oxidation Induction Temperature (OIT) values measured from the DSC first heating curve on color-sorted DKR-324 fractions: Mixed, Color, Transparent, and White. Samples are processed with and without antioxidants to compare the OIT values. A comparison is made on samples post-extrusion (regranulates) in (**a**) and a combination of extrusion and injection molding (dog-bones) in (**b**).

Comparing the effectiveness of AOs in the four batches of DKR-324, as shown in Figure 6, it can be observed that reprocessing the PP with added AOs increases the OIT values. The largest improvement is observed in the White and Color regranulates after extrusion in Figure 6a. For both types of processing, namely extrusion (regranulate) in Figure 6a and extrusion + injection molding (dog-bone) in Figure 6b, the improvement in the OIT upon the addition of AOs is significant. The exception is the Transparent dog-bone sample with AOs, where we observed a large spread in the OIT due to one outlier. However, while comparing the values in Figure 6a directly with Figure 6b, it can also be observed that the OIT values decrease with an additional thermal processing step involving injection molding in all cases. This is in line with expectations as it indicates the consumption of some AOs or the initiation of thermal degradation (if AOs are not present in a sufficient quantity) during the injection-molding step.

Considering the four batches processed without AOs (blue bars in Figure 6), the reductions in OIT values between extrusion (regranulate) and extrusion + injection molding (dog-bone) are 9 °C, 3 °C, 3 °C, and 6 °C, respectively. This slight/moderate reduction, along with the overall OIT values staying above 200 °C, could indicate that most PP waste from packaging possesses residual antioxidants that keep the PP thermally stable for 1–2 reprocessing steps in the recycling process. To understand this further, we also investigated the time needed for the PP to oxidize in an oxygen-rich environment.

**Figure 7:**
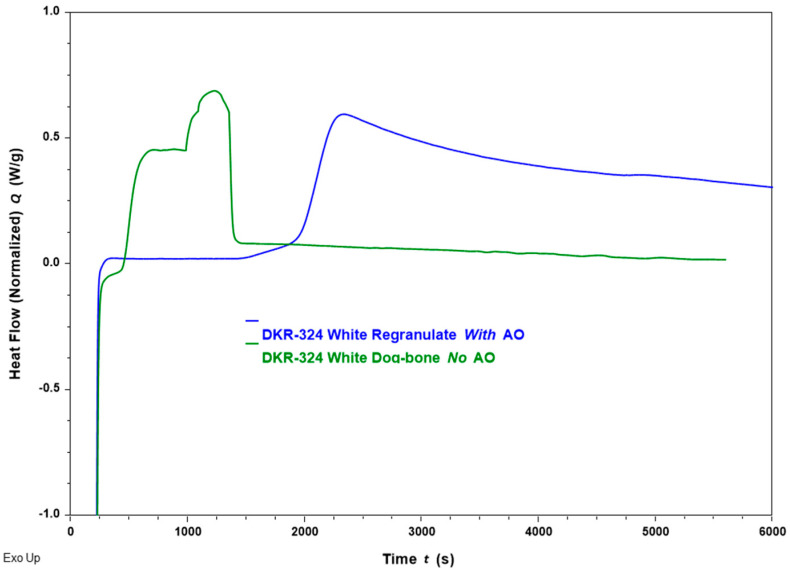
Oxidation Induction time (OIt) values measured from isothermal DSC at 200 °C for DKR-324 White fraction. The OIt value for the regranulate (post-extrusion) is seen to be higher than for the dog bone (post-extrusion and injection molding). Inclusion of additives also delays the OIt. Note that the time in minutes on the x-axis includes the time required for the equilibration and ramp phases of the isothermal tests.

In Figure 7, the time to oxidation is measured by heating the sample from the White fraction in an inert Nitrogen environment up to 200 °C and, subsequently, switching to an oxygen-rich environment. The results plotted in Figure 7 offer insight into the role of AO inclusion, as well as the additional thermal processing step (injection molding of regranulates into dog bones). Unsurprisingly, a lower OIt is observed for the dog bone without AO (green) as it has undergone two thermal processing steps without added stabilization. It also displays two oxidative degradation phases, implying that the residual AO is used up first and the PP rapidly undergoes oxidation later. The samples likely to contain the highest amount of active/intact AO are the Color and White fractions with AOs that have only been extruded (regranulate) based on Figure 6a. In Figure 7, the data for the White regranulate with AO (blue) show a significantly delayed oxidation peak. Furthermore, the oxidation process in the regranulate with AO does not show an end, unlike the other sample. Plenty of intact AO is available to continue this oxidation reaction, and the PP is well-protected against thermal degradation.

A similar comparison is performed on the OIt for all samples post-extrusion, as well as the combination of extrusion and injection molding, and presented in Table 2. The OIt is shown in minutes after subtracting the time required for the ramp and equilibration phases of the test, which last 3.5 min. The samples are also processed with and without antioxidants to compare the OIt values. The lowest OIt values are measured for the Mixed fraction, whereas the inclusion of AO seems to help the Color fraction the most, consistent with the data in Figure 6.

In all cases, the dog-bone samples without AO display the lowest OIt values as they are not thermally protected by intact AO, except the Color fraction. The OIt value of the Color dog-bone sample without AO is almost 37% higher than that of the corresponding regranulate, which is surprising. However, the OIT values in Figure 6a,b also showed some statistical overlap between the regranulate and dog-bone samples of the no-AO Color fraction. The Color regranulate sample also exhibits a tremendously high OIt of 52.5 min when upgraded with AO, quite markedly higher than the other fractions. This could be due to the powder dry-blending approach, which might have caused non-uniform dispersion of the AO, resulting in some samples with extremely high AO levels. It is also possible that the original flakes from the Color fraction themselves possess higher levels of AO, as indicated by an OIT value of ~215 °C, which is slightly higher than other fractions, but this was not studied.

Additionally, the difference in OIt between regranulates that are processed with and without AO is much larger than the same in dog bones. In the White and Color fractions, this alludes to a lot of AO consumption in the injection-molding step. The data for the White and Color fractions in Figure 6a,b show an OIT drop of 10–13 °C between the regranulates and dog bones, adding merit to the above hypothesis. This could be explained by the fact that the White fraction is predominantly made up of injection-molded articles in the waste stream, and typically, injection molding PP grades contain short-chain polymers with more reactive sites for oxidative attack, making them more susceptible to the initiation of thermal degradation during melt processing [42].

In most cases, we observe a two-to-four-fold increase in OIt when the samples are processed with AO. Whether this corresponds directly to the presence of two to four times the amount of AO needs to be studied using HPLC data.

In order to quantify the AO present, HPLC was performed on a limited set of samples. We chose samples with the narrowest and broadest molecular weight distribution for HPLC analysis. The measured weight-averaged molecular weight (Mw) of the white regranulates from SEC was 160 kDa and the Đ was 5. The Mixed fraction, however, displayed a very broad MWD consisting of both extrusion and injection molding grades of PP, and both flexible (small proportion) and rigid PP. The measured Mw of the mixed fraction was measured to be 205 kDa and the Đ was 6. We therefore chose the regranulates of the White and Mixed fractions to study the quantitative tracking of intact and consumed AO. The breakdown of the results from HPLC is organized in Table 3. It should be noted that the samples with AO were processed via powder dry-blending, and we observed some residual powder left in the mixing unit. Therefore, this could explain why the total AO measured between samples (no AO and with AO) from HPLC does not perfectly add up to the expected amounts.

The White fraction mainly consists of injection-molded articles that are likely made up of PP grades with the presence of shorter chains, as mentioned before. It can be seen that the White fraction retains more intact primary AO (Irganox 1010) than the Mixed fraction, possibly because White flakes are typically from consumer packaging with a short life and the primary AOs are not consumed as much. The consumption of the secondary AO (Irgafos 168) is also lower in White originally but not significantly different among the fractions once reprocessed with extra AO. The most important observation, however, is that even in the regranulates where no AO was added, we found residual intact short-term AO (Irgafos 168) after extrusion, over 150 ppm in concentration. This supports our earlier finding from Figure 6 that the recycled PP processed with no AO could still contain residual AO to withstand 1–2 thermal processing steps.

Furthermore, in both Mixed and White fractions, processing the samples with AO led to two to four times more build-up of intact AO. This is observed to be the case for both the primary and secondary AOs. These data were obtained from regranulates, not from samples subjected to an additional injection-molding step. Therefore, whether the antioxidants will persist after the molding process is a question that remains open.

Nevertheless, these data highlight an important question: should we be including additives to recycled polyolefins in the same way we do for virgin materials? It also raises the need to reconsider current strategies to ensure that additive levels are sufficient for processing while remaining safe enough to prevent build-up and the formation of unintended decomposition products. To gain insight into the potential build-up of additives, we also examine closed-loop recycling through multiple cycles and assess the role of additivation during repeated reprocessing via both extrusion and molding.

### 3.2. Closed-Loop Recycling

In this section, the results from the multiple reprocessing of rigid PP in a closed loop are presented.

#### 3.2.1. Composition

The rigid PP (MFR 44 g/10 min) was from back-of-house/kitchen applications in the catering sector that are well suited for closed-loop mechanical recycling. There was no unknown contamination or presence of other polymers such as PE, in this case. The specific application of the rigid PP was in large condiment buckets in catering. However, the hinge of the buckets requires a different grade of PP with a low flexural modulus and high bending strength. The PP grade for the hinge of the bucket had an MFR of 12 g/10 min. It is important to note that in this closed-loop recycling system, the bucket and the hinge could not be separated and were therefore recycled together. Each new bucket made from recycled PP was subsequently attached with a new hinge from virgin plastic. As a result, the amount of hinge-grade PP accumulated over recycling loops ranges from ~3% in Cycle 1 to ~13% in Cycle 5. Table 4 presents the results from SEC on the evolution of MWD in samples from Cycle 1 to 5 processed with antioxidants. Here, a reduction in the number average molecular weight Mn can be seen from Cycles 1 to 4, and a sudden unexpected increase can be seen in Cycle 5. The dispersity is seen to increase until Cycle 4, which could be explained by the consistent accumulation of hinge-grade PP. However, the values of Mn and Đ (Mw/Mn) for Cycle 5 are not consistent with the trend observed.

#### 3.2.2. Oxidative Degradation

**Figure 8:**
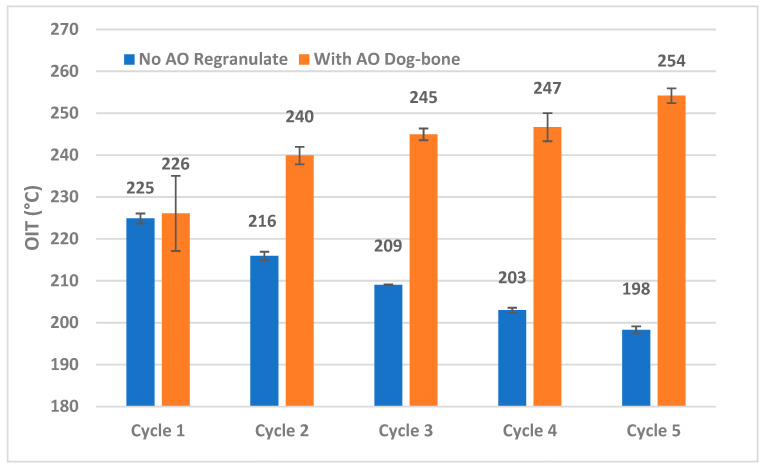
Comparison of OIT on samples through multiple closed-loop recycling with and without the inclusion of antioxidants. The samples that are processed without AO exhibit reduced OIT, which corresponds to the degradation of PP at lower temperatures. The inclusion of antioxidants reveals a delay in the OIT despite an extra thermal processing step, thus enhancing the resistance of PP to oxidative degradation.

An investigation into the DSC heating curves to identify the OIT was carried out in a similar fashion to the open-loop recycling samples. Figure 8 presents the OIT data on PP samples through five cycles of reprocessing with and without AO. The samples with AO (flakes shredded from dog bones) underwent a compounding step followed by an injection-molding step and shredding step, whereas the samples without AO (regranulates) were only reprocessed in the extruder.

The results for the samples without AO show that the OIT steadily decreases from 225 °C to 198 °C, indicating the consumption of all AO and degradation of PP from repeated reprocessing at high temperatures. While processed with added AO, the OIT is pushed to higher values despite an additional thermal molding step. However, in Cycle 1, an overlap in OIT is seen between the regranulates processed without AO and the dog bones that were extruded with AO and subsequently injection molded. The data for dog bones with AO display a large spread, which could imply that the added AO is all consumed in some local mixing areas during the two thermal processing steps. Beyond Cycle 1, increasing OIT values also point to a potential build-up of AO through consecutive cycles of reprocessing. Thus, a quick DSC scan can offer insights into the accumulation of AO for recyclers.

**Figure 9:**
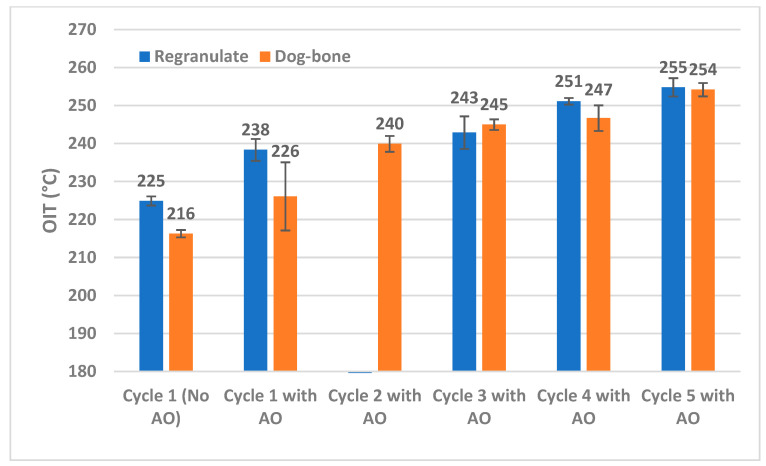
Comparison of OITs on regranulates and dog-bone flakes containing AO through multiple closed-loop recycling. The dog-bone samples underwent extrusion and injection molding, whereas the regranulates only underwent one thermal cycle (extrusion alone).

To further analyze and differentiate the effects of extrusion and remolding, we evaluate samples treated with AO by measuring their OIT values both after extrusion alone and following the injection-molding process into dog-bone samples. These results are presented in Figure 9, benchmarked against data points for Cycle 1 where no AO was added. Note that due to human error, Cycle 2 data could not be collected from this series of extruded regranulates with AO. This, however, does not affect the quality of data from the other Cycles. The results reveal that after Cycle 1, the added antioxidants mitigate the effect of the injection-molding step on oxidative degradation because there is a significant statistical overlap in the data of Cycles 3, 4, and 5 between dog-bone and regranulate samples in Figure 9. Beyond Cycle 2, the injection-molding step does not cause a significant drop in the OIT. Thus, a switchover mechanism after Cycle 1 is observed, where AO is abundantly present. As a result, the availability of these AOs is not limited during injection molding; this thermal step seems to have little impact on propagating further degradation in the PP.

The OIT values in closed-loop samples from the rigid PP are higher overall than those found in the DKR-324 (open-loop) samples. This could be due to a difference in the amount of AO present even at the starting point in Cycle 1 (no AO). In order to measure the Oxidation Induction time, as was done for the DKR-324 samples, isothermal DSC scans were performed. However, for the closed-loop samples, a higher temperature of 225 °C was chosen for the isothermal equilibration step owing to their higher OIT values. Table 5 details the comparison of OIts between samples from each Cycle that were processed with and without AO. The OIt for Cycle 1 regranulates without AO and dog-bones with AO are just 0.6 min apart; this is consistent with the statistical overlap that was observed between these two samples in Figure 8.

It is important to note that the OIT values for Cycles 2–5 with no AO from Figure 8 are below 225 °C. Therefore, it was expected that these samples would immediately undergo thermal decomposition when exposed to air at 225 °C. We observe, however, that Cycles 3–5 with no AO are able to resist oxidation a fraction of a minute in the isothermal state at 225 °C. One possible explanation is that because the heating ramp phase used a high heating rate of 50 °C per minute when the DSC program switched to the isothermal equilibration phase, the actual temperature for the first several seconds may have been below 225 °C, requiring additional time to stabilize. The diffusion of the new purging gas (air) replacing the nitrogen could be another reason for the time delay. Beyond this detail, a clear decreasing trend is observed for the OIt in samples processed through five cycles without AO.

Unsurprisingly, the dog-bone flakes processed with AO after extrusion, molding, and shredding show an increasing trend for OIt. The sample from Cycle 5 was seen to have resisted oxidation for over half an hour at 225 °C, while its measured OIT was 257 °C. These high values of OIt also add evidence to the possibility of AO build-up.

HPLC measurements were performed on samples from Cycle 1 to 5 that were processed with AO in order to break down the intact vs. consumed/degraded AOs. Table 6 shows this breakdown in a similar fashion to DKR-324 samples. Interestingly, we observe a severe build-up of even the secondary AO (meant for depletion in the processing phase) in this case of closed-loop recycling. The primary AO is hardly used through different cycles, which is understandable because the dog bones were shredded immediately and not subjected to any use-phase conditions. Its degradation composition never exceeds 150 ppm.

Another noteworthy aspect of the data is that the amount of degraded secondary AO is lower in Cycle 2 than in Cycle 1. Given that these cycles are processed sequentially, the presence of lower amounts of degraded AO in a Cycle compared to the previous Cycle is unexpected. One possible explanation is that owing to the dry blending of AO, the mixing resulted in an uneven distribution of AO concentrations in the granulates, and choosing a random granulate sample resulted in this anomaly. Another explanation could be that some volatiles (ketones and aldehydes) formed from AO consumption were removed during extrusion in the degassing vent [43]. This result can also be juxtaposed with the data in Table 5 and Figure 9, where a large increase in OIT and OIt was observed for Cycle 2 from Cycle 1 when processed with AOs. However, the increases in OIT and OIt correspond to the amount of intact AO (not degraded AO), which is correctly seen to increase from Cycle 1 to Cycle 2 in Table 6. Subsequently, from Cycle 2 to 5, the amounts of intact secondary AO as well as degraded secondary AO nearly doubled, displaying a linear trend.

These data challenge the standard practice of adding a fixed amount of antioxidants (AOs) during recycling. If we are to ensure food safety for these PP materials, even within a closed-loop system, we must seriously reconsider this approach. The results suggest that a much smaller dose or a more tailored formulation of AOs may be sufficient. Furthermore, whether the amount of AO is adequate can be easily verified through simple DSC scans.

#### 3.2.3. Melt Behavior

**Figure 10:**
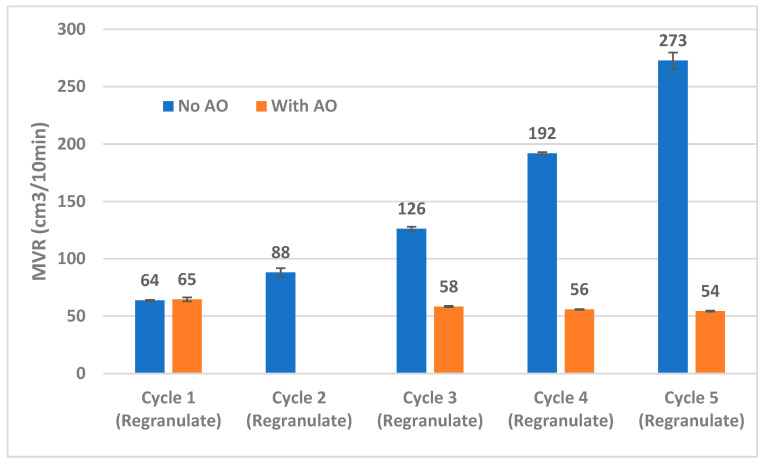
Melt volume rates of the polymer regranulates across multiple loops of recycling in samples processed with and without antioxidants. The trend confirms that the samples processed without AO undergo thermal degradation as seen from an exponentially increasing MVR trend. The slight decrease in the MVR observed for the samples processed with AO can be attributed to the presence of hinge-grade PP (of much lower MFR) in the blend.

Finally, the melt behavior of PP is a key indicator of polymer degradation, which typically occurs through successive chain scission. The melt flow of the PP samples was characterized using an MFR test. However, due to the extremely fast flow of the material, the instrument could not reliably measure the weight of successive cuts. Therefore, Figure 10 presents the melt volume rate (MVR) of the PP samples across successive Cycles instead. Cycle 2 data on regranulates processed with AO is unavailable as noted earlier.

An exponential increase in the MVR was observed when PP samples were reprocessed without AO, indicating rapid chain breakage and thermal degradation of the chains. The PP also became highly amorphous and unprocessable as it approached later Cycles. On the other hand, samples processed with AO maintain their MVR, and in fact, even display a slight decrease in the MVR. While this may be initially surprising, it must be noted that there is an accumulation of hinge-grade PP (with lower MFR) over the Cycles. From the rule of mixtures, it can be surmised that the presence of up to 13% hinge-grade PP in Cycle 5 contributes to this gradual reduction in MVR, calculated to be 53.8 cm^3^/10 min. Additionally, this also supports the data from Table 4 that the chain lengths are somewhat maintained and so is the overall crystallization tendency of the polymer.

#### 3.2.4. Correlation Plots

**Figure 11:**
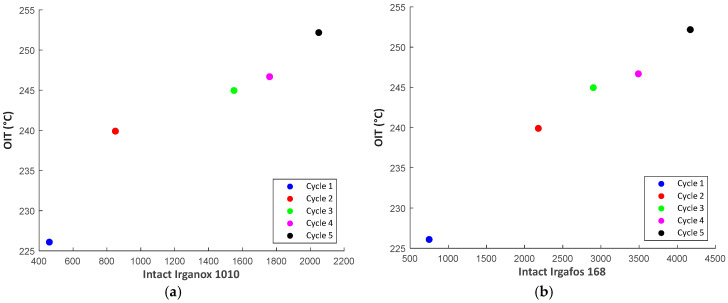
Pair correlation plots between measured variables from DSC and HPLC tests. (**a**) OIT vs. Intact Irganox 1010; (**b**) OIT vs. Intact Irgafos 168. The values of the Oxidation Induction Temperature increase rapidly from Cycle 1 to 2 and subsequently exhibit a limited increase in further cycles of reprocessing. This implies that the amount of AO in Cycle 2 exhibits optimum protection of the PP against thermal degradation.

Figure 11 presents the OIT data from DSC measurements plotted against the measured intact AO quantities from HPLC for the closed loop samples. The result implies two separate linear regimes where, initially, the inclusion of AO improves the OIT significantly above 230 °C. Beyond this addition, the build-up of intact AO is more dominant in comparison to the produced effect on increased OIT. For a recycler, the first linear regime is of interest to determine the optimum addition of AO during the recompounding of PP, which is generally carried out at 230 °C. Overlaying the OIT from a few regranulate samples on their desired processing temperature would reveal if the amount of intact stabilizers present is sufficient to withstand the anticipated processing conditions or whether additional AOs are needed.

## 4. Conclusions

Polypropylene is considered to be a highly suitable material for mechanical recycling, provided it is stabilized with antioxidants to prevent thermo-oxidative degradation during reprocessing and remolding. Our study demonstrates the use of DSC, a simple thermodynamic analysis technique, in evaluating the residual antioxidant levels and determining the necessary additions during recycling. We used DSC measurements to characterize and qualitatively compare residual AO levels in open- and closed-loop recycling of PP. We measured the Oxidation Induction Temperature from DSC sweeps and the Oxidation Induction time under isothermal conditions to estimate and compare the residual antioxidant levels. Our findings revealed a decrease in OIT and OIt between different batches processed with and without the addition of AO, as well as between the regranulates and molded parts.

In open-loop recycling, we observed that the consumption of antioxidants was slightly correlated with the origin of the PP fraction. Variability in the source or origin of different open-loop recycled fractions complicates the comparison of AO levels via OIT between them. However, comparing OIT measurements within a single fraction—processed with and without AO and through multiple thermal processing steps—can offer insights into AO consumption. Surprisingly, intact primary antioxidants persisted in open-loop recycling samples even when no additional AO was added during reprocessing. The findings from closed-loop recycling on a homogeneous PP grade indicated a tendency for significant AO build-up through multiple recycling loops. This accumulation implies that less additional stabilizer is required in closed-loop recycling, which can be advantageous in maintaining the balance between effective stabilization and minimizing potential safety concerns, especially in applications involving sensitive components like food packaging.

These insights from AO quantification and correlations with DSC measurements provide recyclers with valuable guidelines on the optimal quantity of stabilizers needed to ensure the processability and performance of recycled PP. By tailoring the amount of antioxidants based on the specific recycling loop and the initial properties of the PP, recyclers can enhance the efficiency and safety of the recyclates, ultimately contributing to more sustainable and reliable use of recycled PP in various applications.

## Figures and Tables

**Table 1:** MWD of color-sorted DKR-324 fractions. Number-averaged molecular weight (Mn), weight-averaged molecular weight (Mw), and z-averaged molecular weight (Mz) are presented.

Regranulate (Extrusion)	Mn (kDa)	Mw (kDa)	Mz (kDa)	Mw/Mn or Đ
Mixed	34.3	205	640	5.98
Color	30.5	165	520	5.41
Transparent	33.3	185	560	5.55
White	32	162	510	5.06

**Table 2:** Oxidation Induction time values measured from isothermal DSC at 200 °C on mixed and color-sorted DKR-324 fractions.

DKR-324 Type	Oxidation Induction Time (min)			
	Regranulate (Extrusion)		Dog-Bone (Extrusion + Injection Molding)	
	No AO	With AO	No AO	With AO
Mixed	3.3	12.5	2.5	4.7
Color	4.3	52.5	5.9	13.0
Transparent	5.0	11.4	4.0	10.0
White	5.5	29.0	4.0	9.3

**Table 3:** Analysis and breakdown of intact and degraded primary and secondary antioxidants in regranulate samples. The samples were processed with and without the addition of AO during the reprocessing (extrusion) step. Irgafos 168 (secondary AO) is depleted more rapidly during reprocessing than Irganox 1010 (primary AO antioxidant).

Regranulate (Extrusion)	Irganox 1010 Total [ppm]	Irganox 1010 Intact [ppm]	Irganox 1010 Degraded [ppm]	Irgafos 168 Total [ppm]	Irgafos 168 Intact [ppm]	Irgafos 168 Degraded [ppm]
Mixed No AO	190	170	<50	580	150	430
Mixed with AO	470	430	<50	1100	590	510
White No AO	310	220	70	580	190	390
White with AO	510	440	70	1000	500	500

**Table 4:** MWD of regranulates processed with antioxidants across multiple loops of recycling. There is an observed increase in the dispersity of the PP regranulates, which could be due to the accumulation of hinge-grade PP.

Dog-bone (Extrusion + Injection Molding)	Mn (kDa)	Mw (kDa)	Mz (kDa)	Mw/Mn or Đ
Cycle 1 w. AO	36.6	180	650	4.92
Cycle 2 w. AO	31.1	178	620	5.72
Cycle 3 w. AO	30.3	179	600	5.91
Cycle 4 w. AO	29.7	177	570	5.96
Cycle 5 w. AO	37.7	181	570	4.8

**Table 5:** Oxidation Induction time values in minutes measured from isothermal DSC at 225 °C on regranulates through multiple cycles of extrusion. Samples are also processed with and without antioxidants to compare the OIt values. The OIt is gradually reduced when samples are processed without antioxidants and increased in large significant steps when a constant amount of antioxidants is included in each step, indicating a build-up of antioxidants.

Sample	OIt (min)	
	No AO (Regranulates)	With AO (Dog-Bone)
Cycle 1	2.3	2.9
Cycle 2	1.3	7.1
Cycle 3	1.0	9.2
Cycle 4	0.7	21.4
Cycle 5	0.1	36.6

**Table 6:** Analysis and breakdown of intact and degraded primary and secondary antioxidants in the dog-bone flake samples (after extrusion and injection molding in each cycle) through multiple loops of recycling. Similar to the observations in open-loop recycling, the secondary AO is consumed more rapidly than the primary AO. However, in closed-loop recycling, a significant build-up of both types of additives can be observed.

Dog-bone with AO	Irganox 1010 Total [ppm]	Irganox 1010 Intact [ppm]	Irganox 1010 Degraded [ppm]	Irgafos 168 Total [ppm]	Irgafos 168 Intact [ppm]	Irgafos 168 Degraded [ppm]
Cycle 1	500	460	<50	1090	750	340
Cycle 2	890	850	<50	2420	2180	240
Cycle 3	1620	1550	70	3210	2900	310
Cycle 4	1850	1760	80	3870	3490	380
Cycle 5	2210	2050	150	4650	4170	480

## Data Availability

The original contributions presented in this study are included in the article. Further inquiries can be directed to the corresponding author.
